# Health

**Published:** 1879-10

**Authors:** 


### Health

Within a month, a gentleman, whose "house," in a single year cleared six hundred thousand dollars in legitimate business, has been sent to the lunatic asylum, and has since died, at an age but little beyond that at which men are fairly prepared to live to purpose.

Little does the careless, and penniless, and light-hearted passer-by of the splendid palaces of Fifth Avenue, and Union Square, and Fourteenth street, imagine what storms of passion and of fear, what wrecks of heart and hope, what withering of the sweet joys and anticipations of youth, what a drying-up of the better and purer feelings of our nature these stately mansions have sometimes cost their owners.

"What did that house cost you?" is not an infrequent inquiry. "I am ashamed to tell you;" or, "More than it is worth," is a very common response. The true answer in too many instances is, "*It has cost me my soul!*"

To maintain a good name at bank, at the exchange, or on the "street," is an idolatry with many New Yorkers; and to that idol, rather than be sacrificed, men will offer heart, conscience, independence, everything. A good name certainly can never be overvalued; it is worth more than millions of money to the man in business, it is as much his duty as his interest to maintain it at any pecuniary cost, at any personal sacrifice· and it is highly creditable to our business community that so honorable a feeling generally prevails. But the error consists in men placing themselves in positions which present the strongest of all possible temptations to sacrifice independence, and heart, and conscience, in order to maintain their standing in the business world. Beyond all question the great, the most universal error of the age in this country is, the disregard of the scriptural warning against "*hasting to be rich;*" and this neglect brings with it, in multitudes of cases which we never dream of, the *premature* decay of body and mind together, and in the sweeping ruin carries with it down to death, truth, manliness, heart, conscience, all!—confirming the saying, "They that will be rich fall into temptation, and a snare, and into many foolish and hurtful lusts, which drown men in destruction and perdition

